# Society Meetings

**Published:** 1900-11

**Authors:** 


### SOCIETY MEETINGS.

The Medical Union was entertained in the Colonial Parlors of the Genesee, Wednesday evening, October 3, 1900, by Dr. L. B. Dorr, at which time Dr. Ernest Wende read a paper entitled, The recent epidemic of rabies. The attendance was large. Dr. E. Wasdin, Surgeon M. H. S., was present as a guest.

---

The New York State Association of Railway Surgeons will hold its next annual meeting at the Academy of Medicine, New York City, Thursday, November 15, 1900, under the presidency of Dr. J. L. Eddy, of Olean. Dr. C. B. Herrick, of Troy, is the secretary.

THE Esculapian Club was entertained by Dr. C. J. Reynolds, at his home, Thursday evening, October 18, 1900. Dr. W. B. Saltsman read a paper entitled, How to treat urethral stricture. Dr. F. H. Stanbro, of Springville, was present as a guest.

---

THE Mississippi Valley Medical Association will hold its next annual meeting at Put-in-Bay, Ohio, September 10-12, 1901. The officers for the ensuing year are: President, A. H. Cordier, Kansas City, Mo.; vice-presidents, C. F. McGahan, Aiken, S. C., Charles L. Minor, Asheville; secretary, Henry E. Tuley, Louisville, Ky.; treasurer, Dudley S. Reynolds, Louisville, Ky.; chairman of committee on arrangements, J. C. Culbertson, Cincinnati.

---

THE Niagara Falls Academy of Medicine held its regular monthly meeting at the office of Dr. J. H. Miller, Falls Street, on Monday evening, October 1, 1900, at 8.45 p.m. Dr. W. H. Potter read a paper entitled, The uses of boracic acid.

---

THE Buffalo Academy of Medicine held meetings during the month of October, 1900, as follows:

*Section on Surgery.*—Tuesday evening, October 2d. Program: Traumatic insanity, with report of a case trephined twelve years after injury—recovery, Sidney A. Dunham. Discussion by Drs. Henderson and Brownell. Cholelithiasis, Herman E. Hayd. Discussion by E. A. Smith, Roswell Park, and others.

*Section on Medicine.*—Tuesday evening, October 9th. Program: On the involvement of the nervous system in malaria, with a report of a case of malaria presenting the symptoms of disseminated sclerosis, with necropsy and exhibition of slides, W. G. Spiller, professor of nervous diseases, Philadelphia polyclinic. Discussion opened by Charles Cary and James W. Putnam.

*Section on Pathology.*—Tuesday evening, October 16th. Program: An inquiry into the existence of malaria in Buffalo and its environs. Sub-title—Report on mosquito distribution and blood examinations, Irving P. Lyon. Modern theory of the actions of the toxins and antitoxins, G. H. Clowes, Ph. D., Biological Chemist to State Laboratory.

*Section on Ophthalmology, Otology, Rhinology and Laryngology.*—Monday evening, October 22d. Program: The tonsil as a portal of infection, Julius Ullmann. The treatment of chronic tonsilitis, Frederick H. Millener. A case of congenital ocular defect (?) Edmond E. Blaauw.

*Section on Obstetrics.*—Tuesday evening, October 23d. Program: Personal experience with uterine fibroids, H. D. Ingraham.

